# Supplementary material for: Fine Cocoa Fermentation with Selected Lactic Acid Bacteria: Fermentation Performance and Impact on Chocolate Composition and Sensory Properties
Source: Foods. 2023 Jan 11;12(2):340. doi: 10.3390/foods12020340 (PMC9858393; doi:10.3390/foods12020340)
Supplement: Supplementary file 1 [file foods-12-00340-s001.zip › foods-2043191-supplementary.pdf]

Fine cocoa fermentation with selected Lactic Acid Bacteria: fermentation performance and impact on chocolate flavour

Dea Korcari, Alberto Fanton, Giovanni Ricci, Noemi Sofia Rabitti, Monica Laureati, Johannes Hogenboom, Luisa Pellegrino, Davide Emide, Alberto Barbiroli, Maria Grazia Fortina

Supplementary materials

Table S1. List of sensory descriptors used to describe the samples with relevant definitions and reference standards.

Sensory dimension	Sensory Descriptor	Definition	Reference Standard
Appearance	Brown color	Characteristic perceived through the sense of sight referring to the brown color of chocolate	Minimum of the scale (score 1): milk chocolate (Lindt & Sprungli S.p.A.)<br>Maximum of the scale (score 9): 100% criollo chocolate (Esselunga S.p.A)
Odor	Cocoa	Characteristic aroma of cocoa perceived orthonasally with the sense of smell	Cocoa powder Perugina (Nestlé Italiana S.p.A.)
	Red Fruits	Characteristic aroma of red fruits perceived orthonasally with the sense of smell	NeroNero extra dark chocolate with raspberries and almond granules (Novi S.p.A.)
	Roasted	Characteristic aroma of roasted/burnt perceived orthonasally with the sense of smell	100% criollo chocolate (Esselunga S.p.A)
	Honey	Characteristic aroma of honey perceived orthonasally with the sense of smell	Excellence dark with honey and almonds (Lindt & Sprungli S.p.A.)
Taste	Sweet	One of the basic tastes caused by sweet compounds (e.g. sugar) perceived in the oral cavity	Extra dark chocolate Perugina (Nestlé Italiana S.p.A.)
	Bitter	One of the basic tastes caused by bitter compounds (e.g. caffeine) perceived in the oral cavity	100% criollo chocolate (Esselunga S.p.A)
	Sour	One of the basic tastes caused by sour compounds (e.g. lemon) perceived in the oral cavity	100% criollo chocolate (Esselunga S.p.A)
Flavour	Cocoa	Characteristic flavour of cocoa perceived retronasally when swallowing	Cocoa powder Perugina (Nestlé Italiana S.p.A.)

	Red Fruits	Characteristic flavour of red fruit perceived retronasally when swallowing	NeroNero extra dark chocolate with raspberries and almond granules (Novi S.p.A.)
	Roasted	Characteristic aroma of roasted/burnt perceived retronasally when swallowing	100% criollo chocolate (Esselunga S.p.A)
	Honey	Characteristic flavour of honey perceived retronasally when swallowing	Excellence dark with honey and almonds (Lindt & Sprungli S.p.A.)
Mouthfeel sensations	Hardness	Degree of compression between the incisor teeth obtained prior to the breaking of the product	Minimum of the scale (score 1): milk chocolate (Lindt & Sprungli S.p.A.)<br>Maximum of the scale (score 9): 100% criollo chocolate (Esselunga S.p.A)
	Astringency	Tactile sensation of dryness and puckering perceived in the oral cavity	100% criollo chocolate (Esselunga S.p.A)

Table S2. Amounts in ppm of volatile compounds measured from cocoa mass and respective flavour descriptors.

Volatile compounds	Samples						Descriptor
	Control	A	B	C	D	E	
	Volatile compounds (ppm)						
Methyl acetate	0.086	0.085	0.068	0.08	0.104	0.074	Green, fruity, fresh, rum and whiskey-like
2,3-Butandion	1.556	4.631	5.179	1.145	0.824	0.921	Sweet, buttery, creamy, milky
Acetic acid	231.979	276.219	314.842	130.573	67.649	88.608	Pungent, sour, overripe fruit
3-Methyl butanal	1.704	1.582	1.758	1.157	0.645	0.774	Fruity, green, chocolate, nutty, leafy, cocoa
2-Methyl butanal	0.565	0.402	0.629	0.463	0.196	0.211	Musty, rummy, nutty, cereal, caramel, fruity
Pentanal	0.378	0.437	0.541	0.107	0.11	0.257	Winey, bready, cocoa, chocolate notes
Hexanal	0.067	0.208	0.109	0.03	0.037	0.163	Green, woody, apple, grassy, orange
3-Methyl butyric acid	4.46	6.701	4.442	5.633	5.052	4.61	Cheesy, dairy, creamy, sweet, berry
2-Methyl butyric acid	2.403	3.709	2.566	3.605	3.829	3.571	Fruity, acidic, dairy, buttery
2-Heptanone	3.291	9.884	6.016	2.799	1.515	3.413	Cheese, fruity, coconut, waxy, green
2,3-Dimethyl pyrazine	0.066	0.073	0.066	0	0	0	Nutty, nut skin, cocoa, coffee, walnut
Butyrolactone	0.243	0.217	0.203	0.1	0.081	0.074	Milky, creamy with fruity afternotes

Benzaldehyde	0.57	0.733	0.8	0.545	0.419	0.425	Sweet, oily, almond, cherry, nutty, woody
Trimethyl pyrazine	0.402	0.391	0.357	0.345	0.29	0.31	Toasted, nutty, earthy, chocolate, coffee
Benzyl acetaldehyde	0.539	0.177	0.206	0.525	0	0.369	Green, melon, fruity, citrus
Tetramethyl pyrazine	5.22	3.788	2.962	5.926	5.26	3.638	Nutty, musty, cocoa, peanut, coffee notes
2-Nonanone	1.837	4.093	3.061	1.318	1.556	2.213	Cheesy, green, fruity, dairy, dirty, buttery
Nonanal	0.277	0.285	0.354	0.184	0.149	0.158	Citrus, cucumber, and melon rind
2-Phenylethanol	6.088	6.258	7.83	4.771	4.357	4.961	Floral, sweet, rosy and bready
Phenethyl acetate	4.857	3.161	4.248	2.298	1.898	1.843	Sweet, honey, floral, rosy

A: inoculated with *L. fabifermentans* at t_0; B: inoculated with *L. fabifermentans* at t_0 and t_{48}; C: inoculated with *F. rossiae* at t_0; D: inoculated with *F. rossiae* at t_0 and t_{48}; E: inoculated with both strains at t_0 and t_{48}.

Table S3. Effect of samples (6), judges (10), and replicates (2) on the 14 sensory descriptors considered.

Sensory dimension	Sensory Descriptor	Samples	Judges	Replicates	J X S	S X R	J X R
Appearance	Brown color	93.81***	1.83n.s.	0.10n.s.	1.83*	0.57n.s.	1.83n.s.
Odor	Cocoa	13.45***	2.26*	0.00n.s.	1.52n.s.	0.38n.s.	0.75n.s.
	Red Fruits	3.15*	6.84***	3.31n.s.	1.97*	1.39n.s.	0.97n.s.
	Roasted	27.59***	7.05***	1.79n.s.	2.27**	0.62n.s.	1.46n.s.
	Honey	4.97**	1.77n.s.	0.73n.s.	1.18n.s.	0.36n.s.	0.45n.s.
Taste	Sweet	10.08***	2.31*	0.00n.s.	1.45n.s.	2.91*	1.56n.s.
	Bitter	30.31***	8.09***	0.06n.s.	1.14n.s.	1.15n.s.	1.53n.s.
	Sour	15.47***	8.65***	2.06n.s.	1.62n.s.	2.23n.s.	0.47n.s.
Flavour	Cocoa	10.04***	3.71**	0.24n.s.	1.12n.s.	0.24n.s.	1.95n.s.
	Red Fruits	11.25***	5.03***	6.41*	1.40n.s.	1.01n.s.	1.47n.s.
	Roasted	44.90***	2.42*	0.13n.s.	1.03n.s.	0.39n.s.	1.18n.s.
	Honey	33.47***	4.46***	0.06n.s.	1.13n.s.	1.98n.s.	2.01n.s.
Mouthfeel sensations	Hardness	9.85***	4.76***	0.53n.s.	1.49n.s.	0.50n.s.	0.80n.s.
	Astringency	17.30***	11.19***	0.69n.s.	1.16n.s.	0.46n.s.	2.04n.s.

*** significant at p<0.001; ** significant at p<0.01; * significant at p<0.05; n.s. not significant

Table S4. Mean ratings (on a 9-point rating scale) obtained for the 6 dark chocolate samples for each sensory descriptor (*** significant at $p<0.001$; ** significant at $p<0.01$; * significant at $p<0.05$). Different letters by row indicate significant differences ($p<0.05$) according to LSD post hoc test. A: inoculated with *L. fabifermentans* at t0; B: inoculated with *L. fabifermentans* at t_0 and t_{48}; C: inoculated with *F. rossiae* at t_0; D: inoculated with *F. rossiae* at t_0 and t_{48}; E: inoculated with both strains at t_0 and t_{48}.

**Sensory dimension**	**Sensory descriptor**	**Chocolate samples**					
		A	B	C	D	E	Control
Appearance	Brown color***	6.8d	5.2a	6.0c	5.8b	6.0c	7.0e
Odor	Cocoa ***	6.6b	5.9a	6.5b	5.9a	6.4b	6.6b
	Red fruits *	4.5bc	4.5bc	4.6bc	4.4ab	4.7c	4.2a
	Roasted ***	6.4c	5.4a	5.7b	5.6ab	6.2c	6.4c
	Honey **	4.8a	5.5b	4.7a	4.7a	4.5a	4.6a
Taste	Sweet ***	5.4c	5.4c	5.4c	5.5c	4.8a	5.1b
	Bitter ***	5.6c	5.4b	4.8a	4.6a	5.6c	5.6c
	Sour ***	5.6a	5.6a	6.2b	5.6ab	5.6a	6.0b
Flavor	Cocoa ***	6.5b	6.0a	6.3b	6.3b	6.3b	6.8c
	Red fruits ***	4.8c	4.0a	3.8a	3.8a	4.5bc	4.3b
	Roasted ***	6.0b	5.6a	5.6a	6.2c	7.0d	6.8d
	Honey ***	4.0a	5.6d	4.4b	4.8c	3.8a	3.8a
Mouthfeel	Hardness ***	6.4b	6.0a	6.4b	6.3b	6.5b	6.8c
	Astringency ***	4.8a	4.8a	4.8a	5.5b	5.3b	4.9a

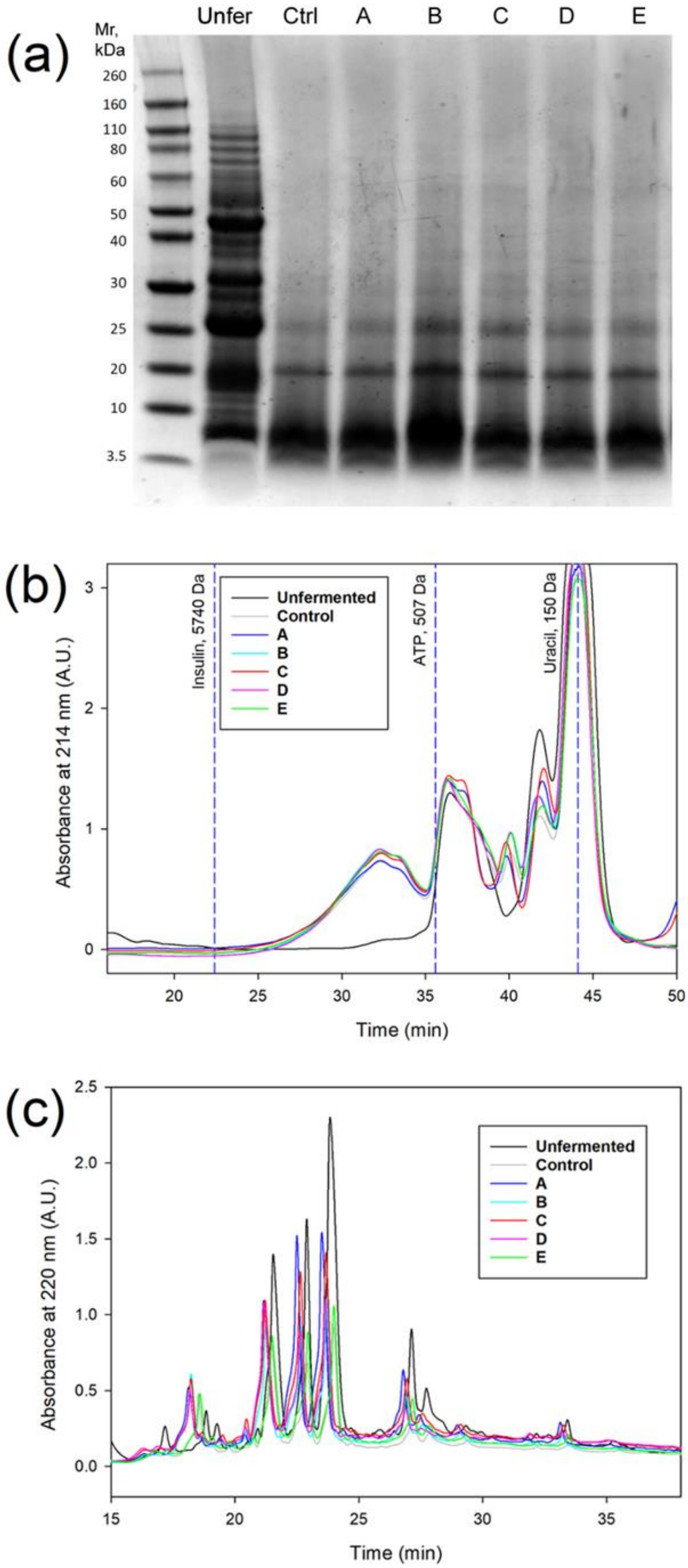
(a)
Unfer
Ctrl
A
B
C
D
E
Mr, kDa
260
160
110
80
60
50
40
30
25
20
10
3.5
(b)
Insulin, 5740 Da
ATP, 507 Da
Uracil, 150 Da
Unfermented
Control
A
B
C
D
E
Absorbance at 214 nm (A.U.)
Time (min)
(c)
Unfermented
Control
A
B
C
D
E
Absorbance at 220 nm (A.U.)
Time (min)

Figure S1. Protein and peptide pattern of unfermented and fermented samples. (a): SDS-PAGE of total protein extracts; Unfer: unfermented cocoa beans; Ctrl: spontaneous fermentation; A: inoculated with *L. fabifermentans* at t_0; B: inoculated with *L. fabifermentans* at t_0 and t_{48}; C: inoculated with *F. rossiae* at t_0; D: inoculated with *F. rossiae* at t_0 and t_{48}; E: inoculated with both strains at t_0 and t_{48}. (b): SE-HPLC chromatograms of peptide extracted from cocoa beans. (c): RP-HPLC chromatograms of peptide extracted from cocoa beans.
